# Integrated geophysical healthy assessment for eco development and coastal sustainability in Ras Gamila, Egypt

**DOI:** 10.1038/s41598-025-26234-3

**Published:** 2025-11-23

**Authors:** Alhussein Adham Basheer, Zamzam M. A. Darwish, Abdelnasser Mohamed, Adel Diab Mohammed Kotb

**Affiliations:** 1 Geology Department, Faculty of Science, Helwan University, Ain Helwan, Cairo, 11795 Egypt; 2 Qena Faculty of Arts, South Valley University, Qena, Egypt; 3 National Research Institute of Astronomy and Geophysics (NRIAG), Helwan, Cairo, 11421 Egypt

**Keywords:** Geophysical survey, Coastal sustainability, Seawater intrusion, Radon analysis, Multi-criteria decision analysis, Sustainable development, Climate sciences, Environmental sciences, Hydrology, Planetary science, Solid Earth sciences

## Abstract

Arid coastal regions like Ras Gamila, Egypt, face pressing environmental challenges, including seawater intrusion, freshwater scarcity, and development pressures, which threaten their ecological and economic sustainability. This study bridges a critical research gap by developing an integrated framework that links subsurface geophysical stability with surface environmental conditions to guide eco-development. We employed a multi-method approach, combining vertical electrical sounding (VES), time-domain electromagnetic (TDEM) soundings, shallow seismic reflection, soil radon analysis, and spatial data from digital elevation models (DEMs), shoreline dynamics, and climatological factors. Our results delineate a critical freshwater-bearing Nubian sandstone aquifer (19–94 m thick, resistivity: 228–302.5 Ωm) and identify significant seawater intrusion (resistivity: 1.1–2.5 Ωm). A novel sustainability matrix, integrating these diverse datasets, classifies the region into three distinct zones: high-sustainability inland areas (35–45% of the region) suitable for immediate development, moderate-sustainability central zones (35–45%) requiring targeted improvements, and low-sustainability coastal areas (25–30%) necessitating restoration and protection. The findings provide a scalable, geophysically-informed model for sustainable planning in arid coasts, directly supporting United Nations Sustainable Development Goals (SDGs) 6 (Clean Water) and 13 (Climate Action) by offering a science-based strategy for balancing economic growth with environmental conservation.

## Introduction

**Fig. 1:**
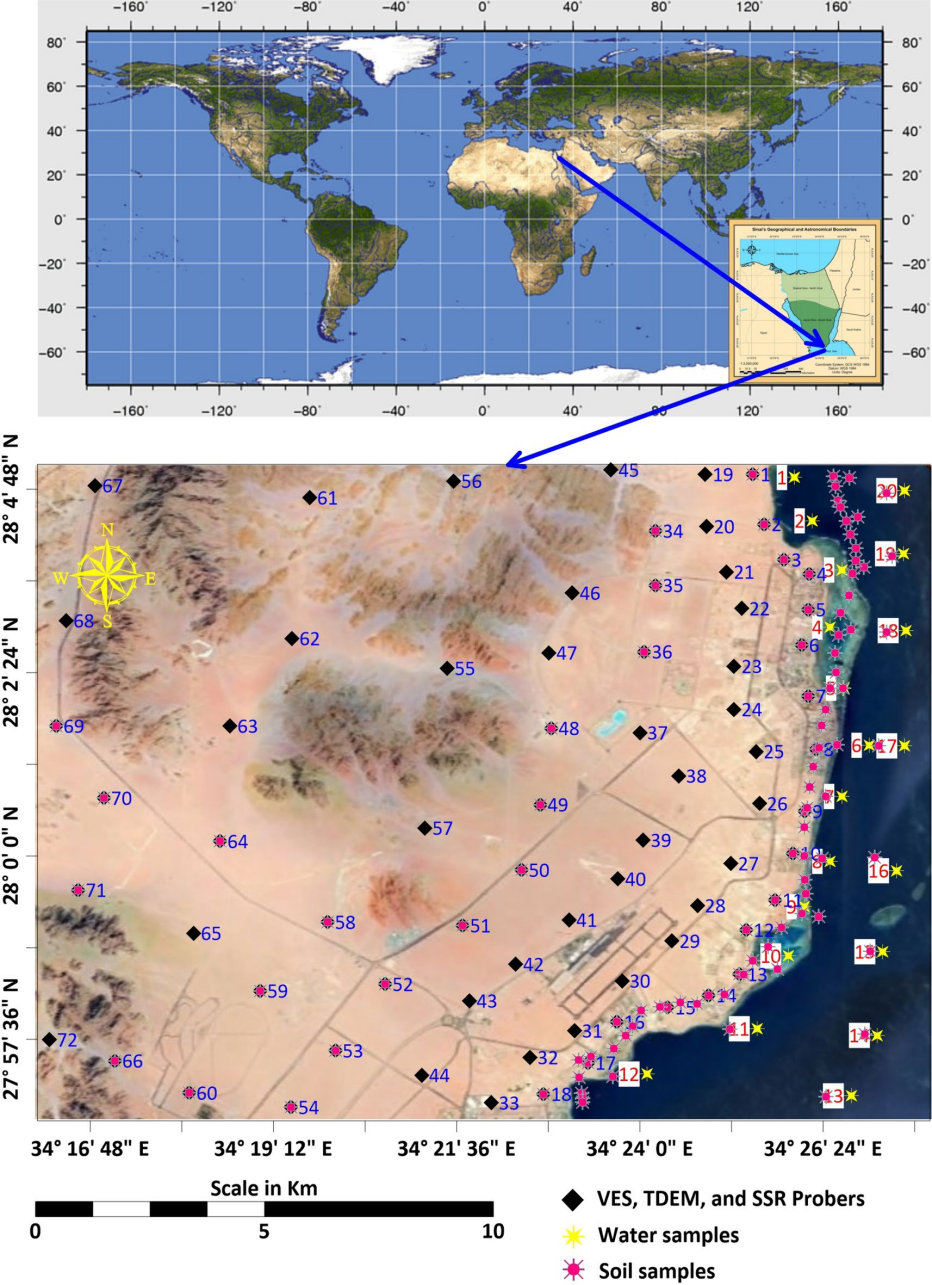
Location map of the study area. Base satellite imagery: Copernicus Sentinel-2 (European Space Agency; https://scihub.copernicus.eu, accessed 20 August 2024), and Landsat 9 (U.S. Geological Survey Earth Explorer; https://earthexplorer.usgs.gov, accessed 20 August 2024). Map compiled and annotated by the authors using ArcGIS Pro (v3.2; https://www.esri.com/software/arcgis-pro) and Surfer (v25; https://www.goldensoftware.com/).

The purpose of this study is to provide an integrated analysis of the environmental and geophysical characteristics of Ras Gamila to guide sustainable development strategies^1^. Our work directly supports UN SDGs 6 (clean water) and 13 (climate action). The study area lies between latitudes 27° 56′ 32.05″ and 28° 5′ 6.49″ in the North and longitudes 34° 27′ 48.01″ and 34° 16′ 6.50″ in the East (Fig. 1). Specifically, the research focuses on combining geophysical data, including Vertical Electrical Sounding (VES) and Time Domain Electromagnetic (TDEM) soundings, with environmental data to develop a holistic understanding of the area. Seawater intrusion into freshwater aquifers is a significant concern, requiring careful monitoring of salinity levels and groundwater reserves^2,3^. By combining these diverse datasets, the study aims to establish a model for sustainable planning that minimizes environmental degradation while promoting development. This comprehensive approach ensures that future projects in Ras Gamila can maintain a balance between economic growth and ecological preservation.

The assessment of sustainability in coastal arid regions necessitates a focus on the subsurface conditions that dictate freshwater availability and construction viability. In this study, key subsurface parameters—such as aquifer salinity, depth to bedrock, and groundwater elevation—are derived primarily through geophysical methods. For instance, Electrical Resistivity Tomography (ERT) is employed to delineate saltwater intrusion interfaces and map freshwater aquifers, while shallow seismic reflection surveys provide critical data on bedrock depth and subsurface geology. These geophysically-derived parameters serve as direct proxies for quantifying ‘aquifer vulnerability’ and ‘substrate stability,’ which are fundamental to the region’s long-term sustainability^4^. This approach allows us to move beyond superficial assessments and base our sustainability evaluation on the measured stability of the foundational resources.

Coastal zones globally are under immense stress from climate change, urbanization, and resource exploitation. In arid regions, these pressures are exacerbated by water scarcity and fragile ecosystems, making sustainable development a complex challenge^5^. While numerous studies have examined individual aspects of coastal zones, such as seawater intrusion, geotechnical stability, or tourism impacts, integrated assessments that quantitatively link subsurface geophysical conditions with surface ecological health and climatic dynamics remain rare^3^. This gap is particularly pronounced in the Red Sea region, where pristine environments like Ras Gamila coexist with intense development ambitions. Our study addresses this by introducing a holistic sustainability matrix that quantifies the interactions between subsurface hydrology (e.g., aquifer salinity, bedrock depth) and surface climatical dynamics (e.g., humidity, rainfall).

Ras Gamila is located just north of Sharm El Sheikh, which is a region of immense environmental sensitivity, where the Red Sea’s coral reefs and marine life form some of the most biodiverse ecosystems in the world. The area’s environmental significance is well known, yet it faces increasing development pressures due to its global reputation as a tourist hub (Fig. 1). These pressures present numerous challenges, particularly regarding land use, waste management, and the impact of tourism on fragile ecosystems. Given these factors, geophysical measurements are critical for understanding soil conditions, groundwater reserves, and the potential for seawater intrusion, which can significantly impact local ecosystems. Geophysical data also help in identifying areas prone to erosion, enabling better management of coastal infrastructure and protecting valuable tourist resources. Sustainable development in Sharm El Sheikh and its surroundings requires a detailed understanding of the subsurface environment, ensuring that future developments are resilient and environmentally friendly^6^.

Existing studies linking geophysical and environmental data have shown that coastal regions like Ras Gamila face unique challenges due to the dynamic interplay between land and sea. For instance, the integration of geophysical surveys with marine environmental parameters has proven successful in identifying zones of seawater intrusion, subsurface freshwater aquifers, and areas vulnerable to coastal erosion. Previous research in the Red Sea and similar coastal zones has demonstrated the importance of monitoring parameters in mitigating the impact of coastal development. Studies have also emphasized the need to assess geophysical soil characteristics to understand the stability of coastal regions, which is vital for building resilient infrastructure^7^. By linking environmental factors with geophysical data, researchers can develop a more comprehensive approach to managing the delicate ecosystems in these coastal areas, ensuring that development is both sustainable and environmentally conscious^8^.

This study adopts an integrated geophysical approach to address three key challenges facing the sustainable development of Ras Gamila: first, seawater intrusion dynamics using VES and TDEM resistivity analysis to determine the extent of saltwater intrusion into freshwater aquifers and evaluate mitigation strategies such as managed aquifer recharge. Second, geophysical environmental interactions by analyzing how soil resistivity, aquifer depth, and bedrock topography interact with climatic stresses to constrain development potential. Third, health risks are associated with radon by integrating soil radon measurements with subsurface models to assess exposure risks and guide land use zoning for sustainable infrastructure planning.

By combining these datasets into a predictive sustainability matrix, the ability of geophysical-based planning will be tested to reconcile economic growth and resource conservation in arid coastal areas. This methodology will provide a viable framework for regions facing similar hydrogeological and environmental stresses.

## Geological setting

**Fig. 2:**
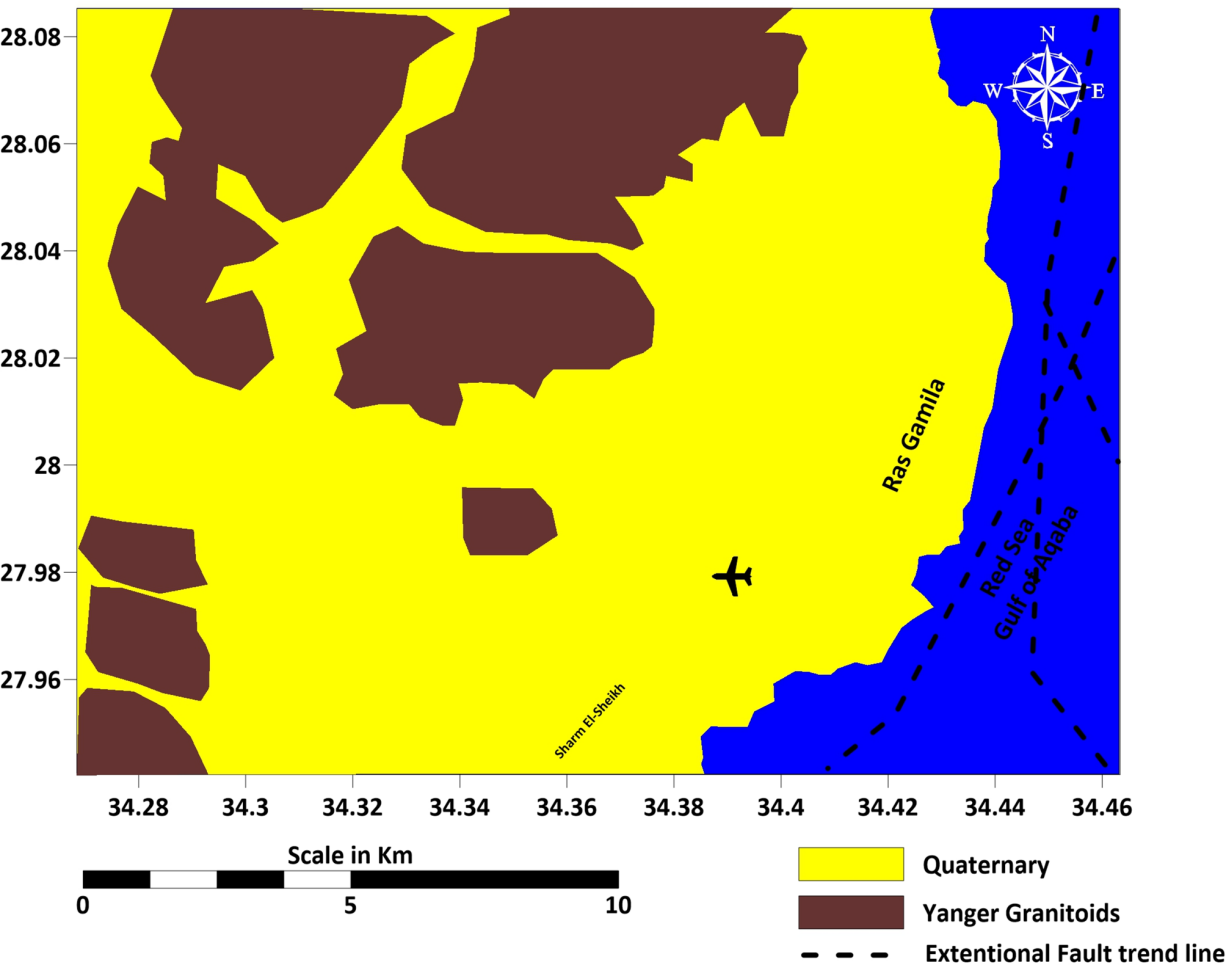
Geology map of the study area. Geological boundaries compiled from published data and field mapping by (https://www.esa.gov.eg), overlain on ESRI World Imagery (ESRI, USGS; https://www.arcgis.com/home/item.html?id=10df2279f9684e4a9f6a7f08febac2a9, accessed 20 August 2024). Map created and modified by the authors using ArcGIS Pro (v3.2; https://www.esri.com/software/arcgis-pro) and Surfer (v25; https://www.goldensoftware.com/).

The Ras Gamila area in South Sinai, Egypt, showcases a diverse and complex geological and tectonic setting, indicative of extensive tectonic processes and stratigraphic evolution. Figure 2 presents the geological map of Ras Gamila and its surrounding areas, illustrating the different rock formations, fault lines, and sedimentary layers. It highlights the geological complexity of the region, including various lithological units. The region’s geology spans from Precambrian basement rocks, part of the Arabian Nubian Shield, to more recent Pliocene and Quaternary deposits. Precambrian rocks, primarily granites, gneisses, and schists, were shaped during the Pan African orogeny, with subsequent intrusions and tectonic deformations reflecting the area’s dynamic geological history. These basement rocks, along with basaltic dikes and rift structures associated with the Red Sea and Gulf of Aqaba, provide insights into the tectonic evolution of the region, dating back approximately 950 to 450 million years^9–11^.

Overlying the basement rocks are sedimentary formations ranging from the Paleozoic to the Cenozoic, including sandstones, shales, and limestones. The Miocene epoch, particularly with formations like South Gharib and Abu Dabbab, marks the onset of the Red Sea Rift, shaping the area’s stratigraphy. Quaternary deposits, including coral reef terraces and alluvial sediments, reflect ongoing tectonic activity and sea-level changes, emphasizing the region’s active sedimentary and tectonic processes^9,10,12,13^.

Structurally, the area is influenced by major fault systems, primarily linked to the Red Sea opening and the Gulf of Aqaba’s formation. These fault systems, part of the Dead Sea Transform Fault, exhibit both strike-slip and normal fault movements, leading to block-tilted structures and significant tectonic uplift. This tectonic complexity influences the landscape, resource distribution, and groundwater systems, with faults controlling groundwater flow in fractured basement rocks and porous sedimentary layers (Fig. 2)^9,10,14–17^.

Economically, Ras Gamila holds potential for mineral resources such as copper, gold, and phosphate, which are often associated with hydrothermal processes along fault zones. Offshore, the area presents opportunities for oil and gas exploration, making it a region of economic and scientific interest. However, challenges in resource management and environmental conservation arise due to the intricate relationship between its tectonic and hydrogeological systems. Balancing development with the preservation of natural resources is essential for sustainable progress in the region. Understanding geology is critical for sustainable planning as it influences land use, water infiltration, and the overall stability of construction activities^18–23^.

## Materials and methods

**Fig. 3:**
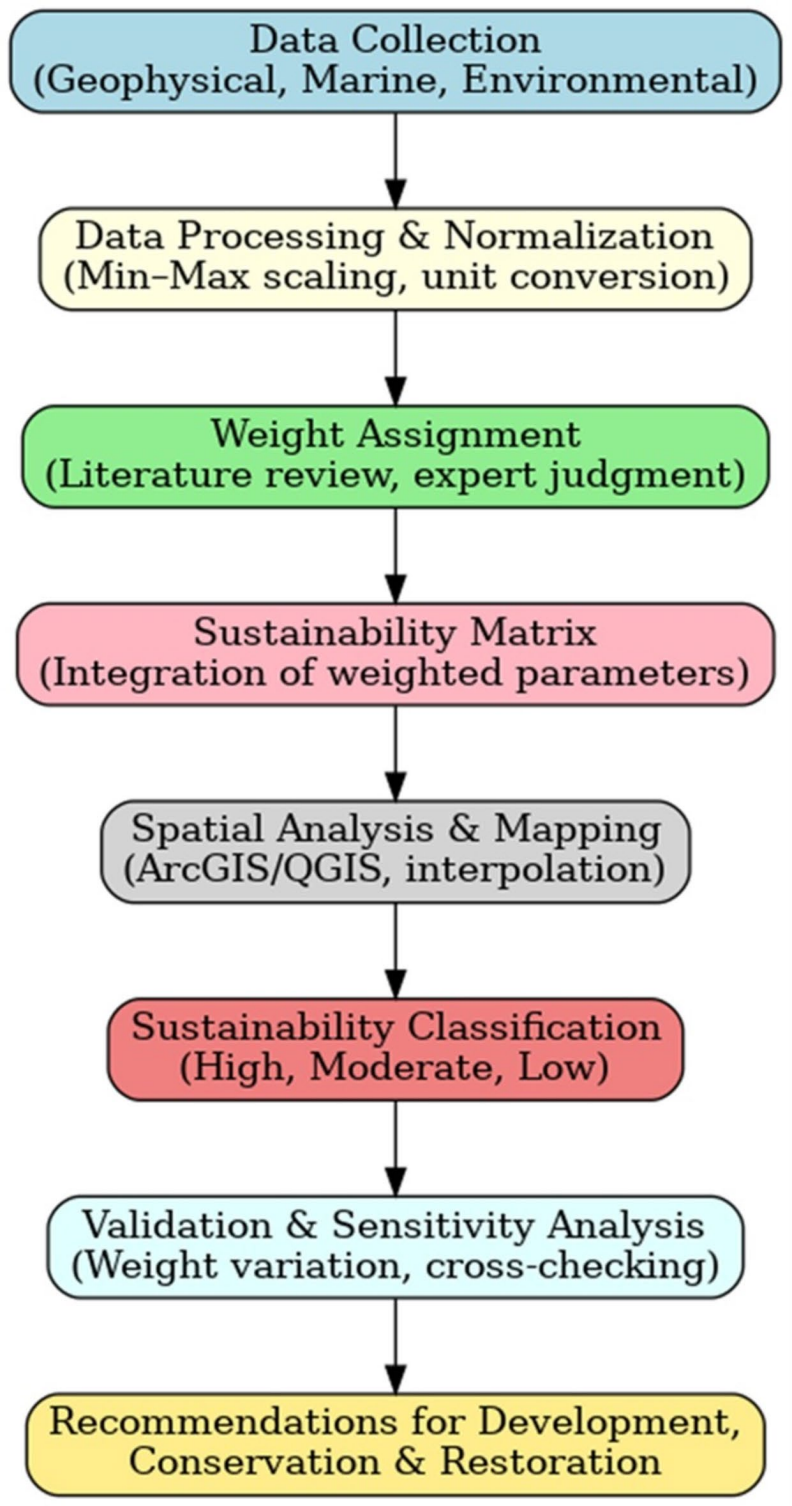
Workflow chart (sequence brief). Chart created by the corresponding author.

This study employed an integrated, multi-disciplinary methodology to assess the sustainability of the Ras Gamila area. The workflow, summarized in Fig. 3, involved four primary phases: (1) extensive field data collection across geophysical, geochemical, and environmental domains; (2) data processing and interpretation using specialized software; (3) spatial analysis and integration within a Geographic Information System (GIS) environment; and (4) the development and application of a sustainability evaluation matrix to synthesize all findings into a coherent zoning plan.

### Digital elevation model (DEM)

The DEM was generated using LiDAR data from the Egyptian Survey Authority (2023) and 21 ASTER GDEM v3 data^24^, supplemented by ground control points collected with differential GPS (Topcon HIPER VR) and a Topcon Total Station. Preprocessing steps, including noise removal, point cloud classification, georeferencing, and interpolation, were performed using^25,26^ software. The DEM was used to calculate slope and aspect, delineate watersheds, and assess terrain stability and flood risks^27–31^.

### Watershed and drainage network delineation

Watershed and drainage delineation were performed using preprocessed DEM data. Flow direction was calculated using the D8 and D∞ algorithms, followed by flow accumulation analysis to identify stream channels^32,33^. The Strahler stream order method classified the streams, and pour points were used for watershed delineation^26,34,35^ software facilitated these analyses, aiding in applications such as flood prediction, water resource management, and erosion modeling.

### Shoreline characteristics

Remote sensing data, including satellite imagery and aerial photographs, were used to classify shoreline types and assess changes over time. Field surveys validated findings and gathered data on beach profiles, sediment grain size, and vegetation^36^. GIS tools integrated remote sensing and field data, allowing for spatial analysis of shoreline dynamics. Statistical analysis correlated shoreline characteristics with factors like wave action and human activities^37,38^.

### Humidity

To characterize the spatiotemporal variability of relative humidity, a network of hygrometers (Model^39^ was installed across urban, forest, and coastal zones. All sensors were calibrated and operated in strict adherence to the manufacturer’s specifications and^40^ guidelines. This monitoring campaign captured high-resolution data on diurnal and seasonal cycles, with concomitant measurements of temperature, air pressure, and wind speed to provide a comprehensive atmospheric profile. The resultant dataset was subjected to rigorous statistical analysis to elucidate the interdependence between humidity and co-varying meteorological parameters. These relationships are subsequently visualized and discussed through a series of graphical representations, including temporal humidity trends and multivariate comparative charts^41,42^.

### Rainfall data

During the years 2019–2024, rainfall data for the South Sinai region were collected using a combination of ground-based and remote sensing methods. Ground-based observations were conducted with standard rain gauges (such as tipping-bucket and weighing rain gauges) installed at meteorological stations across the area, ensuring precise measurement of precipitation totals and intensities. These data were complemented by satellite-based observations (e.g., GPM and TRMM), providing spatially distributed estimates of rainfall, especially in remote or mountainous zones where access is limited. The combined use of direct measurement devices and satellite data allowed for robust monitoring and characterization of the temporal and spatial variations of precipitation across South Sinai throughout the 2019–2024 period^43–45^.

### Geophysical survey

#### Vertical electrical sounding (VES)

A total of 72 VES soundings were conducted using^46^ equipment with a maximum electrode spacing of 1000 meters. The Schlumberger array was used, and resistivity data were processed with the^47^ software to interpret subsurface variations. (VES) was employed to characterize subsurface resistivity variations, providing valuable insights into the depth and extent of groundwater-bearing formations, which are essential for sustainable groundwater management^48–50^.

#### Time domain electromagnetic sounding (TDEM)

A total of 72 TDEM soundings were performed using EM37^51^ equipment with a 100 m^2^ loop. Decay curves were processed with the^52^ software to develop resistivity models. VES and TDEM data were combined for a comprehensive subsurface analysis using^53,54^ software. (TDEM) method complemented the resistivity surveys by capturing lateral conductivity changes with higher sensitivity, thereby improving the accuracy of groundwater reservoir delineation and reducing uncertainty in depth estimation^9,48,55^.

#### Shallow seismic reflection

A survey utilizing 72 shallow seismic reflection profiles was conducted using a^56^ instrument to determine the depth to bedrock and map subsurface geological features. The method involved deploying 24 geophones (14Hz) spaced 10 meters apart along each profile, with seismic waves generated by a sledgehammer reflecting off subsurface layers. The recorded travel times were processed using advanced software, such as^57,58^.

### Soil sample analysis for radon concentration

A total of 56 soil samples (18 samples from shoreline, and 18 samples over the study area) have been collected and analyzed by using three detectors of^59–61^ to analyze the energy peaks of the emitted radiation^62–64^.

### Sustainability matrix development

The sustainability matrix was constructed using a Multi-Criteria Decision Analysis (MCDA) approach, specifically the Analytical Hierarchy Process (AHP)^65^, which is widely used in environmental and land-use planning.

#### Parameter selection and weight assignment

Fifteen parameters were selected based on their relevance to coastal sustainability and data availability. They were grouped into three categories: Environmental Quality (EQ), Resource Potential (RP), and Geophysical Factors (GF). Weights were assigned to each parameter through a combination of the AHP and expert judgment. A pairwise comparison matrix was constructed where experts evaluated the relative importance of each parameter concerning the overall goal of sustainable development. Parameters posing direct risks to human health (e.g., radon concentration) or representing fundamental constraints on development (e.g., freshwater availability, bedrock stability) were assigned higher weights. The consistency ratio (CR) of the pairwise comparison was calculated to be 0.08, which is below the threshold of 0.1, indicating a satisfactory consistency in judgment^65^. The normalized weights are presented in Table 2.

#### Normalization and index calculation

Parameters were normalized to a scale of 0 to 1 using the linear min-max method to make them dimensionless and comparable:

1 $$Normalized\;value = \frac{{X_{max} - X_{min} }}{{X - X_{min} }}$$

where (X) is the original value of the parameter. For cost factors (e.g., seawater intrusion thickness, where higher values are worse), the formula was inverted (1 - Normalized Value), and *X*_min_​ and *X*_max_​ are the minimum and maximum values observed for that parameter across the study area.

The sustainability score (S) for each zone was then calculated using the weighted sum method:

2 $$S = \mathop \sum \limits_{i = 1}^{n} \left( {W_{i} \times X_{i} } \right)$$

where (w_i_) is the normalized weight of the i-th parameter, and (x_i_) is its normalized value.

#### Sensitivity analysis

**Fig. 4:**
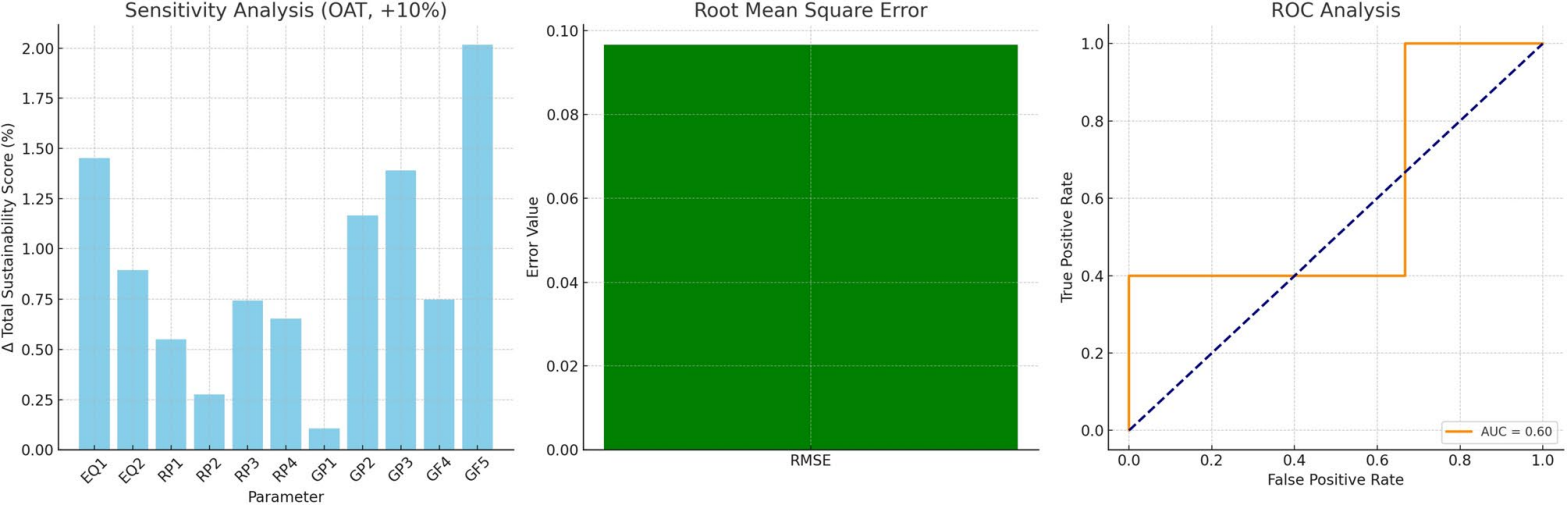
Left: One-at-a-time (OAT) sensitivity analysis (+ 10% perturbation per parameter); Middle: Root-mean-square error (RMSE); Right: Receiver operating characteristic (ROC) curve with area under the curve (AUC). Plots generated by the corresponding author.

A sensitivity analysis was performed to evaluate the robustness of the sustainability index and identify the parameters that most influence the output. The One-At-A-Time (OAT) method was employed, whereby the weight of a single parameter was varied by ± 10% and ± 20% while adjusting the weights of the others proportionally to maintain a sum of 1. The resulting change in the total sustainability score was recorded and analyzed^66^ (Fig. 4).

Table 1 shows the summary of the category, data sources, software, and application for data collection**.**

**Table 1:** Summary of the data collection category.

Category	Data sources	Software	Applications
Land data			
(DEM)	LiDAR, 21 ASTER Satellite, Ground Measurements (GPS, Total Station), Jason3 2025	Global Mapper, Surfer	Topography Analysis, Hydrological Modeling
Watershed delineation	DEM, ArcGIS, MODIS 2025, Surfer Software	ArcGIS, Surfer	Flood Prediction, Water Resource Management
Shoreline characteristics	Satellite Imagery, Field Surveys, GIS, NESDIS 2025, NOAA 2025. NCEI 2025	Remote Sensing Software, GIS	Coastal Planning, Erosion Studies
Rainfall	Tipping-bucket & weighing rain gauges (local meteorological stations)	MS Excel, GIS	Analyzing daily, monthly, and annual rainfall totals, Flood risk assessment, climate trend studies, water resources planning
Geophysical survey (VES, TDEM & SSR)	Syscal Pro (VES), EM37 Geonics (TDEM), StrataVisor NZXP	IPI2Win, ZondEM2D, IX1D, ZondRes2D, SeisImager, ReflexW	Subsurface Resistivity, Groundwater Studies, bedrock level
Soil analysis for radon concentration	HighPurity Germanium (HPGe), a NaI(Tl) scintillation, alpha spectroscopy	ORTEC	Radon concentration in soil, Annual Effective Dose, Surface Exhalation Rate, Mass Exhalation Rate

## Results

**Fig. 5:**
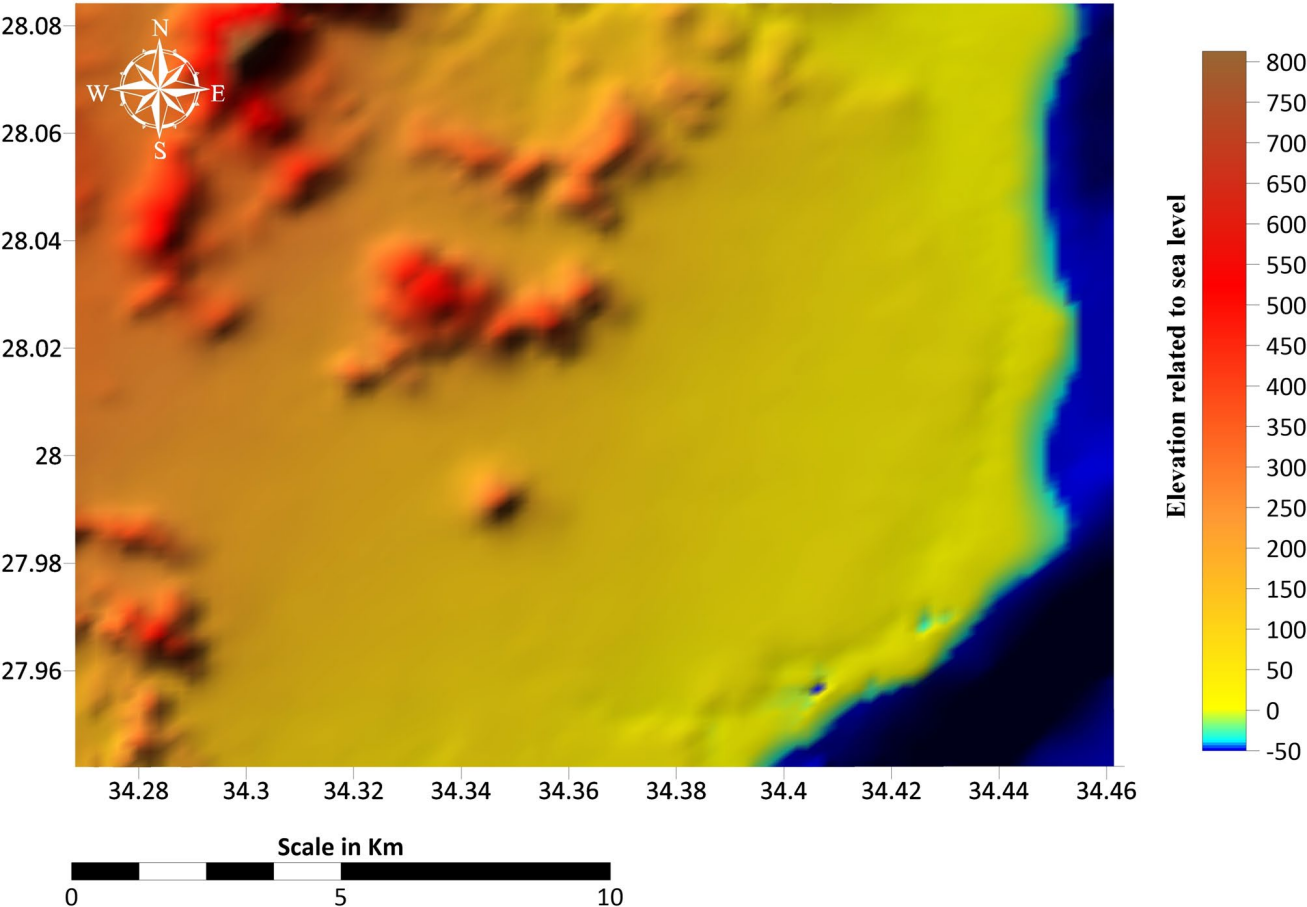
Digital Elevation Model (DEM) of the study area. DEM data: SRTM 30 m (NASA, USGS; accessed 20 August 2024 from https://earthexplorer.usgs.gov). Map produced by the corresponding author using ArcGIS Pro (v3.2; https://www.esri.com/software/arcgis-pro) and Surfer (v25; https://www.goldensoftware.com/).

The DEM map (Fig. 5) depicts the topographical variation in Ras Gamila and nearby regions, showcasing highland, lowland, coastal shoreline, and seafloor bathymetry around areas. Elevation data is essential in understanding water flow, erosion patterns, and potential areas for human development. It can also help identify flood-prone areas and areas suitable for infrastructure development or agriculture. Figure 5 presents the elevation data of Ras Gamila, with altitudes ranging from near sea level to elevated plateaus and mountainous areas. The lowest elevations are located in the sea at 1600 meters below sea level, and the moderately lower elevation is along the coast. In comparison, higher elevations of about 896 meters above sea level, associated with tectonic uplift, are inland.

**Fig. 6:**
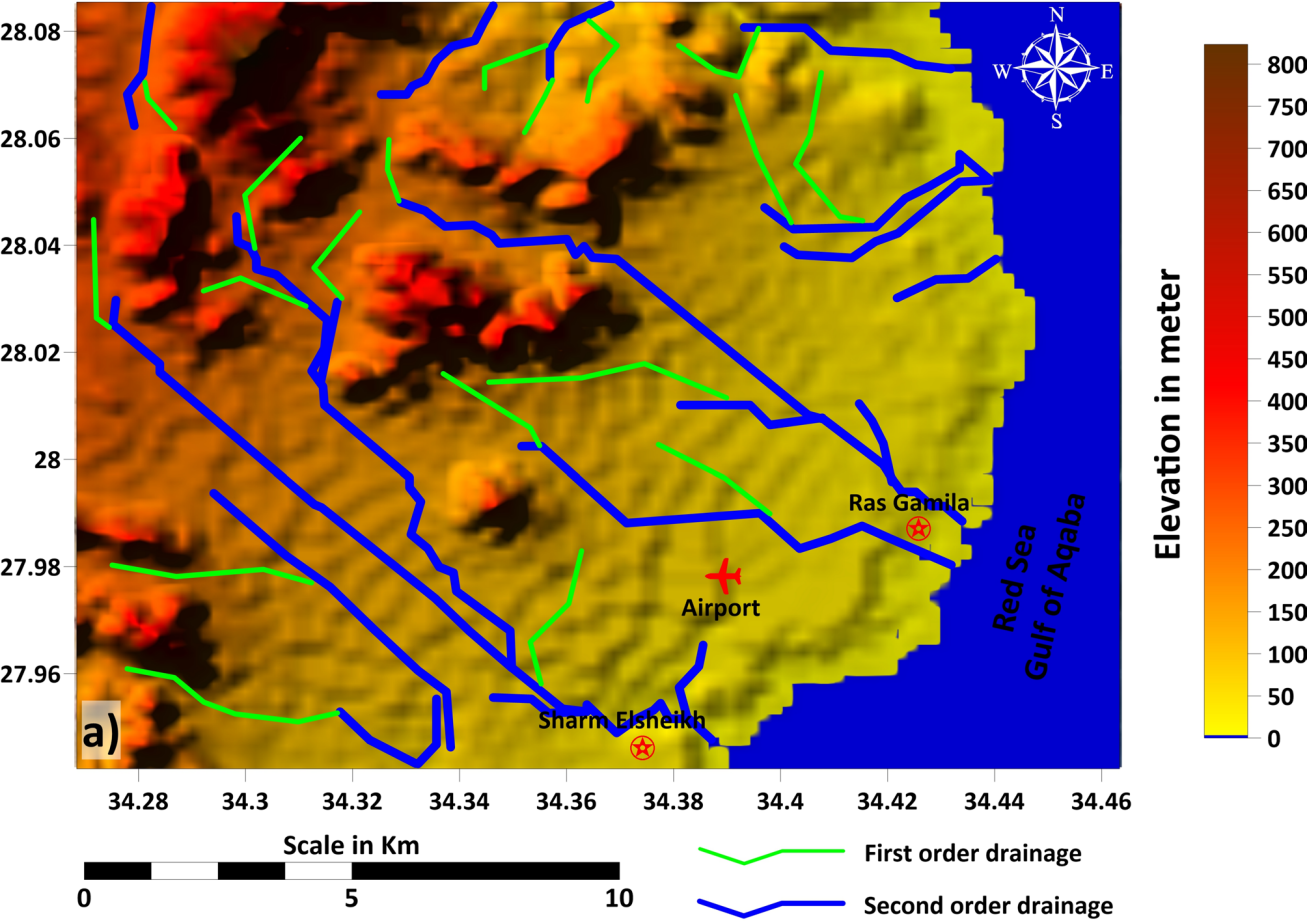
Natural drainage system of the study area, derived from DEM data (SRTM 30 m, NASA, USGS; accessed 20 August 2024). Map generated by the corresponding author using ArcGIS Pro (v3.2; https://www.esri.com/software/arcgis-pro) and Surfer (v25; https://www.goldensoftware.com/).

The natural drainage systems and wadis in the Ras Gamila area are vital for surface water flow and indicate the role of tectonic and geomorphological features in shaping hydrological patterns. Eighteen drainages of the first order are located in the north and east parts, while nine drainages of the second order (Fig. 6). The shoreline dynamics within the Sharm El Sheikh and Ras Gamil area are not isolated coastal processes but are intrinsically governed by regional hydrology and geomorphology, detailed in Figs. 5 and 6. The analysis reveals a complex and dynamic coastline that can be classified into three primary types based on dominant processes and observable trends: erosional headlands, stable embayments, and accretionary wadi mouths. The steep topographic gradient from the mountainous hinterland to the coast, as shown in Fig. 6, is the primary driver of these dynamics, as it controls the energy and sediment supply delivered to the shoreline by ephemeral drainage systems (wadis). Erosional headlands are characterized by exposed rocky outcrops and cliffs, where wave energy is concentrated, leading to active backshore erosion and minimal sediment accumulation; these areas show a long-term trend of gradual retreat, threatening any proximal infrastructure. In contrast, stable embayments, often nestled between headlands, exhibit pocket beaches with a relative balance between sediment input and wave-driven transport, resulting in minor, seasonal fluctuations but overall mid-term stability; these areas are highly valued for tourism but remain vulnerable to changes in sediment supply.

The most dynamic classification is found at accretionary wadi mouths, where second-order drainage channels, clearly delineated in red in both figures, discharge their water and sediment load during flash flood events. These zones are characterized by prograding shorelines, built from alluvial fans and deltaic deposits, and exhibit a net trend of accretion over time. However, this trend is highly episodic and contingent on the frequency and magnitude of rainfall events in the upland catchments. A critical finding of this analysis is the anthropogenic disruption to these natural dynamics. Coastal infrastructure, such as roads and resorts built along the low-lying coastal plain, often obstructs these natural drainage pathways^67^, preventing sediment from reaching the beach and starving downdrift areas, leading to unintended erosion. Furthermore, the intense development in areas like Sharm El Sheikh, frequently located in the stable embayments and low-elevation zones identified as high-risk in Figs. 5 and 6, has armored the shoreline with seawalls and revetments. While protecting assets in the short term, this hardening often exacerbates erosion elsewhere by reflecting wave energy and disrupting alongshore sediment transport. Consequently, the shoreline’s evolution must be understood as a direct function of inland hydrological processes, where the protection of first-order stream catchments from upland erosion is as crucial for coastal sustainability as managing direct coastal impacts, aligning with^68^ recommendations for integrated coastal zone management that builds resilience against future climate stressors.

The shoreline analysis of the Sharm El-Sheikh area and Ras Gamila, located along the Gulf of Aqaba in the Red Sea, highlights the critical interplay between coastal topography, drainage patterns, and potential coastal processes. This relationship is fundamental to understanding the dynamics and vulnerabilities of these distinct coastal zones^69^. The shoreline near Sharm El-Sheikh is characterized by a low-lying coastal plain, with the surrounding terrain gradually rising inland. This relatively flat topography is incised by second-order drainage channels that flow toward the coast, indicating natural pathways for surface runoff and sediment transport^70^. The morphology of this drainage network suggests that the coastal zone is episodically influenced by fluvial processes, where rainfall events can lead to sediment deposition and localized erosion along the shore. While this topography provides an advantageous setting for urban infrastructure, such as the nearby airport, it also necessitates careful consideration of flood risks from these drainage channels discharging near developed areas^71–73^.

In contrast, the shoreline at Ras Gamila features a more rugged and elevated coastal landscape. First- and second-order drainage lines converge steeply toward the coast, suggesting that runoff from the higher inland terrain reaches the shoreline with greater energy. This higher-energy discharge has a more pronounced impact on shoreline erosion and sediment transport patterns, creating a dynamic and potentially sensitive environment^74^. The complex interaction between hillslope runoff and coastal processes at Ras Gamila likely plays a significant role in shaping sedimentary features and influencing the stability of adjacent coastal habitats, such as coral reefs or seagrass beds^75^.

It is clearly noted that the coastal dynamics of both the Sharm El-Sheikh and Ras Gamila shorelines are fundamentally controlled by their respective drainage basins and local topography. The identified drainage networks are critical for delivering both runoff water and sediments to the coast, which in turn influences erosion and accretion patterns. Understanding this intricate relationship is essential for effective coastal zone management, including strategies for mitigating erosion, protecting infrastructure, and conserving sensitive marine ecosystems. Proactive monitoring and management of these drainage-shoreline interactions are paramount for enhancing the region’s resilience against coastal hazards, particularly those associated with episodic rainfall and flash flood events in arid environments^76^. This integrated analysis provides a valuable foundation for guiding sustainable development and environmental protection efforts in these ecologically and economically significant areas.

**Fig. 7:**
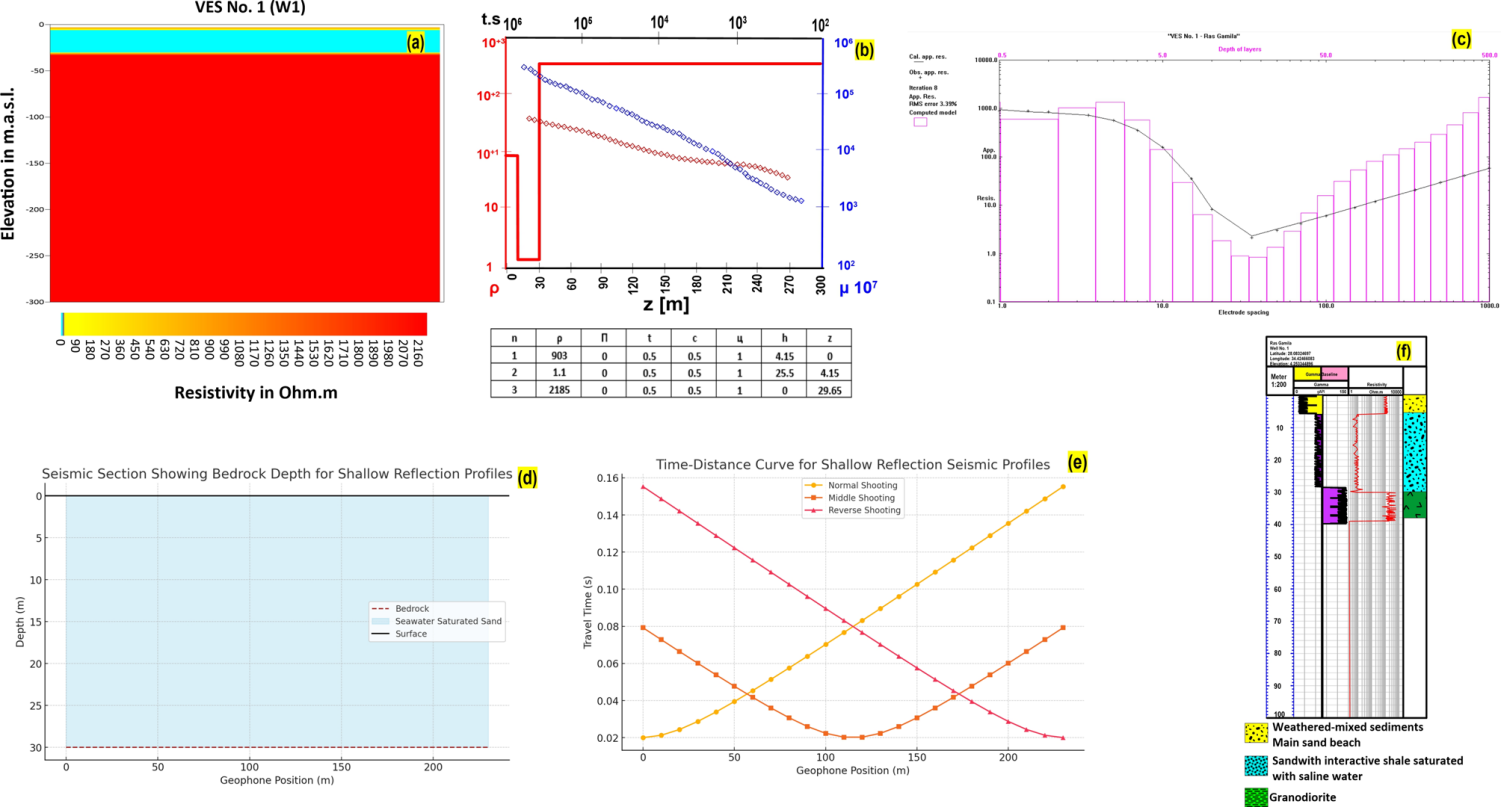
(**a**) Example of resistivity vertical distribution (probe No.1); (**b**) TDEM interpretation (probe No.1); (**c**) VES sounding (probe No.1); (**d**) Shallow seismic reflection profile No.1; (**e**) Time–Distance curve of shallow seismic reflection profile No.1; (**f**) Well-log at probe No.1 location. Figures created by the corresponding author using IPI2Win (http://geophys01.geol.msu.ru/rec_lab3.htm), ZondTEM1D (http://zond-geo.com/english/zond-software/electromagnetic-sounding/zondtem1d/), ReflexW (https://www.sandmeiergeo.de/reflexw.html), SeisImager (https://www.geometrics.com/software/seisimagersw), and SeisViewer (https://wiki.seg.org/wiki/Dictionary:Seisviewer).

**Fig. 8:**
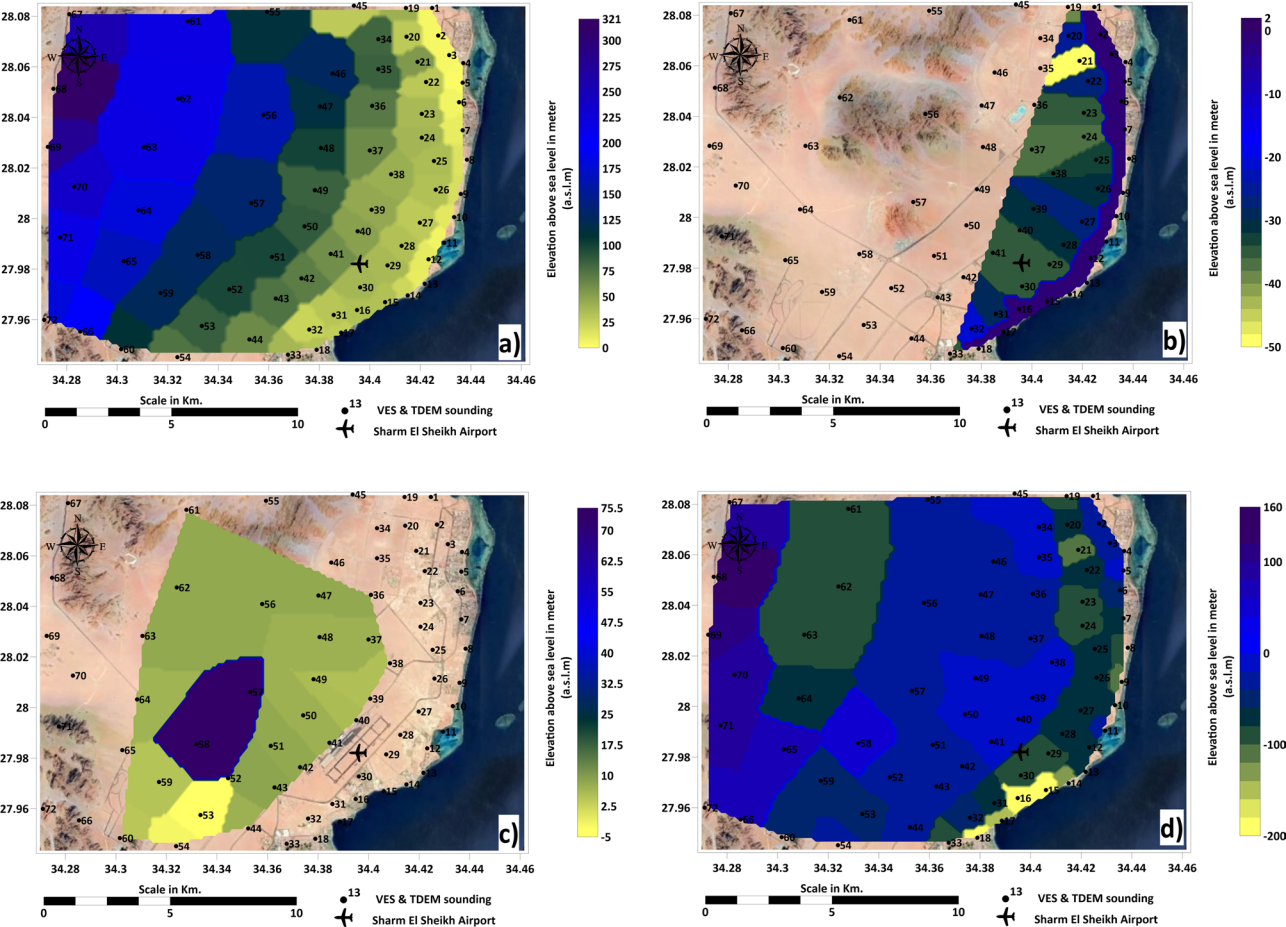
(**a**) Elevation of surface geoelectrical layer; (**b**) Elevation of second geoelectrical layer; (**c**) Elevation of third geoelectrical layer; (**d**) Elevation of fourth geoelectrical layer. Maps generated by the corresponding author using ArcGIS Pro (v3.2; https://www.esri.com/software/arcgis-pro) and Surfer (v25; https://www.goldensoftware.com/)., using DEM data (SRTM 30 m, NASA, USGS; accessed 20 August 2024).

**Fig. 9:**
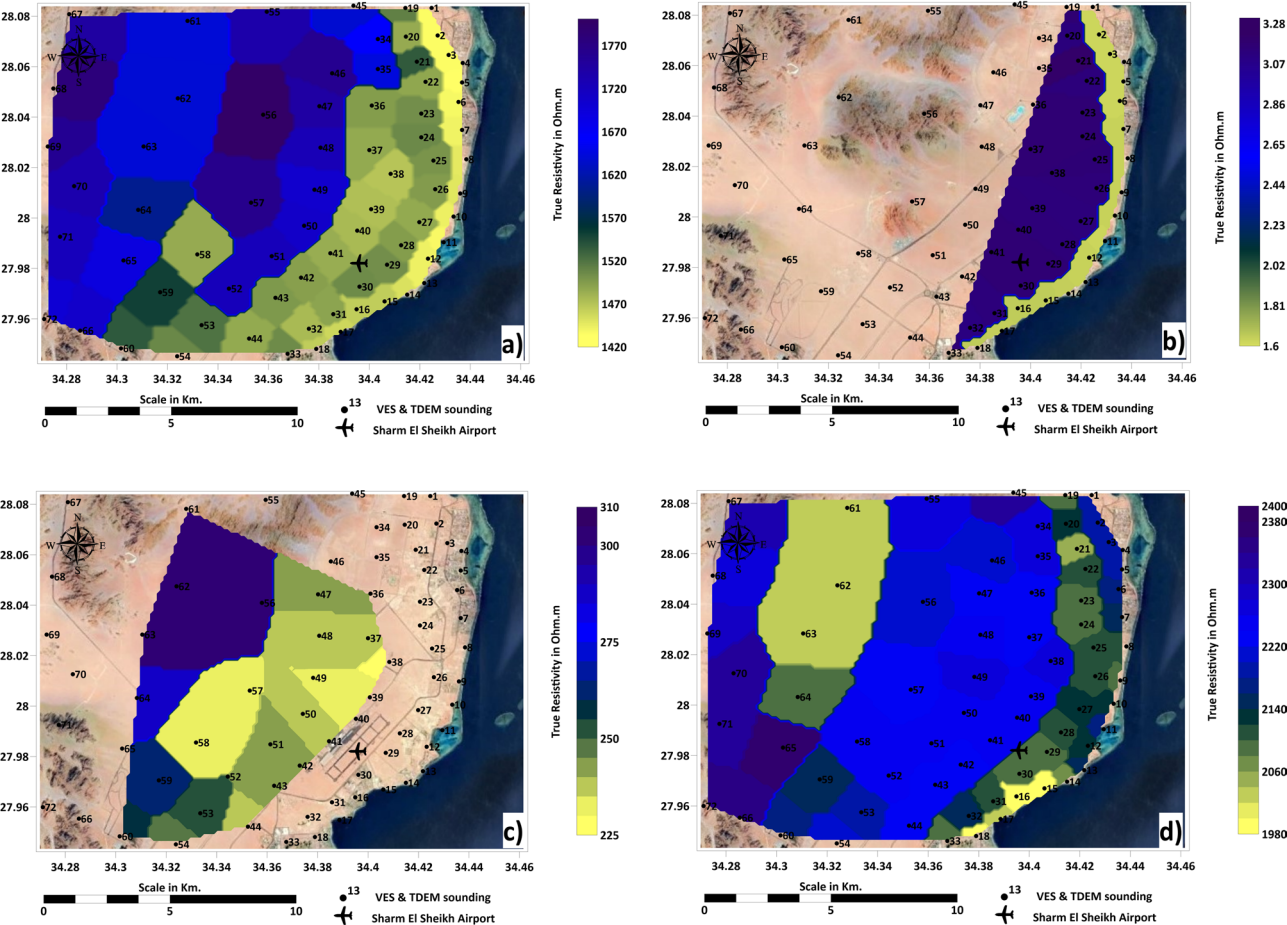
Distribution of resistivity values for (**a**) surface geoelectrical layer; (**b**) second layer; (c) third layer; (**d**) fourth layer. Maps created by the corresponding author using ArcGIS Pro (v3.2; https://www.esri.com/software/arcgis-pro) and Surfer (v25; https://www.goldensoftware.com/).

An example of geophysical interpretations supported with well logs charts is shown in Fig. 7a–f. The geophysical interpretation in Fig. 8 illustrates four distinct geo-electrical layers critical to understanding the subsurface structure of the Ras Gamila area. Figure 8a highlights the elevation of the surface layer, composed of Quaternary Sands, which forms the uppermost part of the geo-electrical profile with a thickness range from 2.5 meters and resistivity of 902 Ohm.m near the shoreline to 210 and resistivity of 1812 Ohm.m in the inside plateaus. This layer is crucial for determining soil permeability and potential for land use in infrastructure projects. Figure 8b focuses on the second layer, consisting of sand with seawater intrusion, a key factor in assessing groundwater salinity issues. Even though this layer is located near the shoreline (thickness varies between 14 to 194 meters and resistivity of 1.1 to 2.5 Ohm.m), the presence of seawater encroaching into this layer poses a threat to freshwater availability and could affect agricultural and drinking water supplies. Figure 8c reveals the third layer, which consists of Nubian sandstone containing fresh water. This layer represents a vital resource for sustainable freshwater extraction and its protection is essential for longterm water security in the region with thickness ranges between 19 to 94 meters and resistivity of 228 to 302.5 Ohm m. Figure 8d examines the deeper fourth layer, composed of younger Granodiorites, which holds potential for resource exploration, including the possibility of mineral extraction or geothermal energy utilization, and its elevation varies from 121 to 194 meters related to sea level and resistivity of 1987 to 2367.5 Ohm.m. These layers offer a comprehensive view of the subsurface conditions and their implications for environmental management and resource exploitation. Figure 9a–d shows the distributions of resistivity values of the four geo-electrical layers over the study area.

Applying the shallow seismic reflection method in the same locations as previous geophysical probes, specifically (VES) and (TDEM) surveys, has proven effective in determining the depth of bedrock across the study area. This method facilitated the creation of a detailed contour map that illustrates the spatial distribution of bedrock depths. The results derived from seismic reflection are consistent with those obtained from electrical and electromagnetic methods, underscoring the complementary nature of these techniques^77^. Furthermore, the seismic data were integrated with information from regional maps and previous studies, allowing for a more comprehensive understanding of subsurface geological structures^78–81^.

**Fig. 10:**
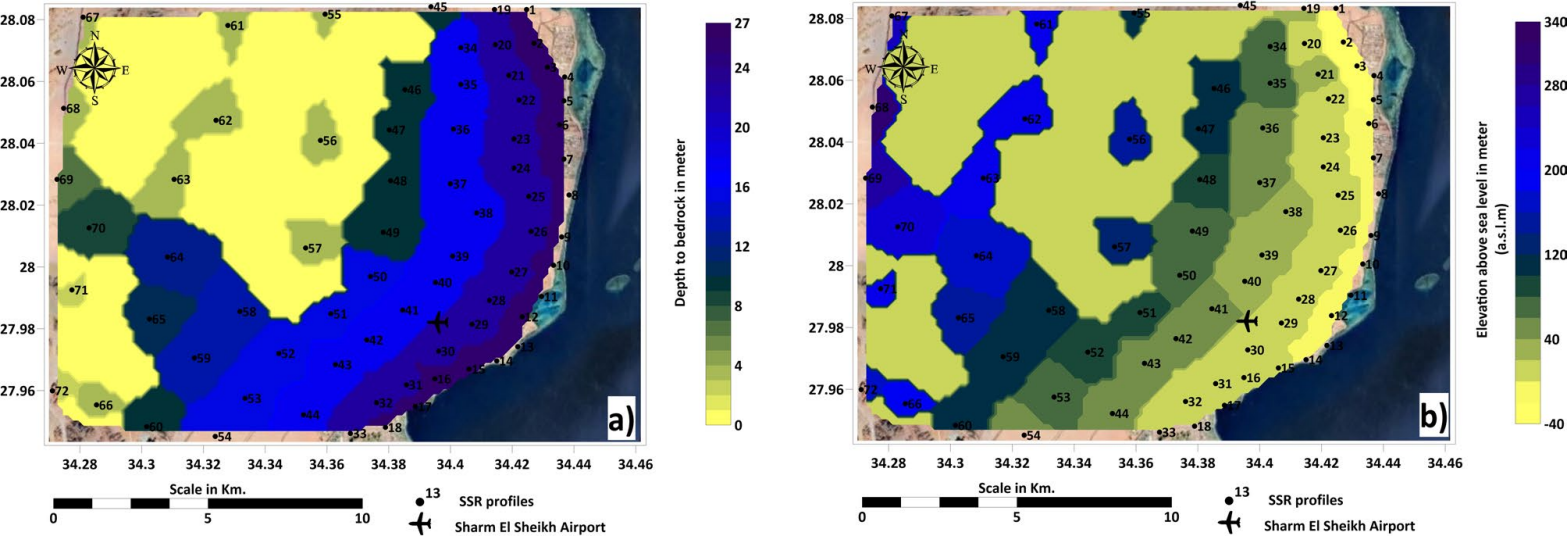
(**a**) Distribution of bedrock depth; (**b**) Distribution of bedrock elevation. Maps generated by the corresponding author using ArcGIS Pro (v3.2; https://www.esri.com/software/arcgis-pro) and Surfer (v25; https://www.goldensoftware.com/)., based on USGS DEM (SRTM 30 m; accessed 20 August 2024) and geophysical interpretations.

The bedrock depths in the study area exhibit significant variation, ranging from approximately 122 meters above sea level in regions near the mountains and elevated terrain to as low as 195 meters below sea level towards the coastal areas. This pronounced gradient reflects the transition from higher elevations close to the mountainous regions to lower elevations near the beach. The depth and elevation results of the bedrock map provide critical insights into the geological framework of the area, which is essential for a range of geotechnical, environmental, and hydrological investigations (Fig. 10a, b).

The dataset of soil samples presents measurements of Radon concentration, annual effective dose, surface exhalation rate, and mass exhalation rate in the investigation area.

**Fig. 11:**
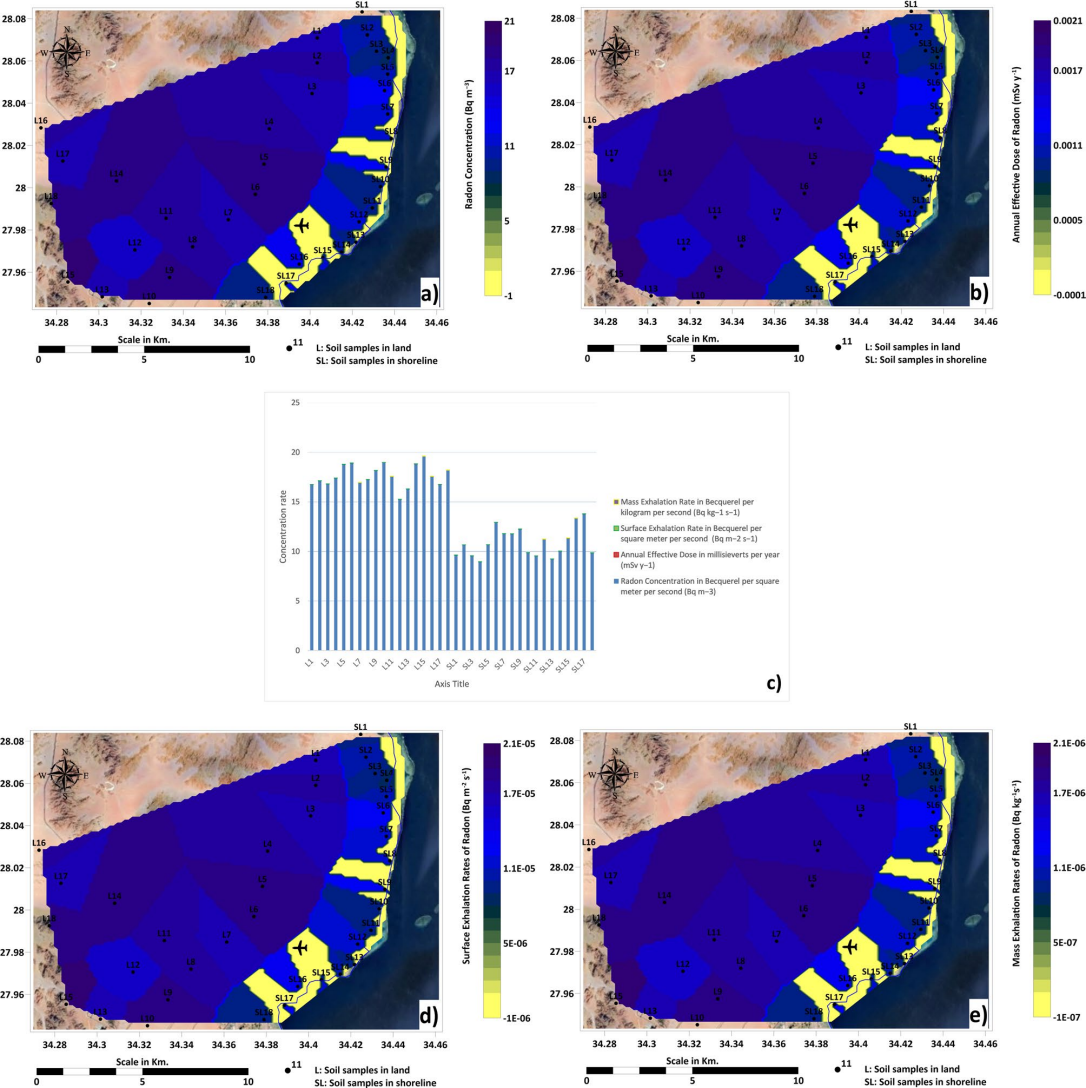
(**a**) Radon concentration (Bq m⁻^3^); (**b**) Annual effective dose (mSv y⁻^1^); (**c**) Radial multi-graph of radon data; (**d**) Surface exhalation rate (Bq m⁻^2^ s⁻^1^); (**e**) Mass exhalation rate (Bq kg⁻^1^ s⁻^1^). Maps generated by the corresponding author using ArcGIS Pro (v3.2; https://www.esri.com/software/arcgis-pro) and Surfer (v25; https://www.goldensoftware.com/).

Inland samples, the radon concentration ranges from approximately 15.25 Becquerel per square meter per second (Bq m^3^) to 19.61 Bq m^3^ (Fig. 11a,c), and the annual effective dose is generally between 0.00152 in millisieverts per year (mSv y^1^) and 0.00196 mSv y^1^ (Fig. 11b,c). Surface exhalation rates lie between 1.525 × 10^5^ in Becquerel per square meter per second (Bq m^2^ s^1^) and 1.960 × 10^5^ Bq m^2^ s^1^ (Fig. 11c,d). The mass exhalation rates are similar within the range of 1.525 × 10^6^ Becquerel per kilogram per second (Bq kg^1^ s^1^) to 1.961 × 10^6^ Bq kg^1^ s^1^ (Fig. 9c,e). In shoreline soil samples, Radon concentrations in this area range between 8.96 and 13.79 Bq m^3^ (Fig. 9a,c), and the annual effective dose spans from 0.00089 to 0.00138 mSv y^1^ (Fig. 11b,c). Surface exhalation rates are between 8.961 × 106 and 1.379 × 105 Bq m^2^ s^1^ (Fig. 11c,d) Mass exhalation rates vary from 8.961 × 10^7^ to 1.379 × 10^6^ Bq kg^1^ s^1^ (Fig. 11c,e). The shoreline area displays intermediate radon concentrations, with values generally lower than peak values in the inland area.

**Fig. 12:**
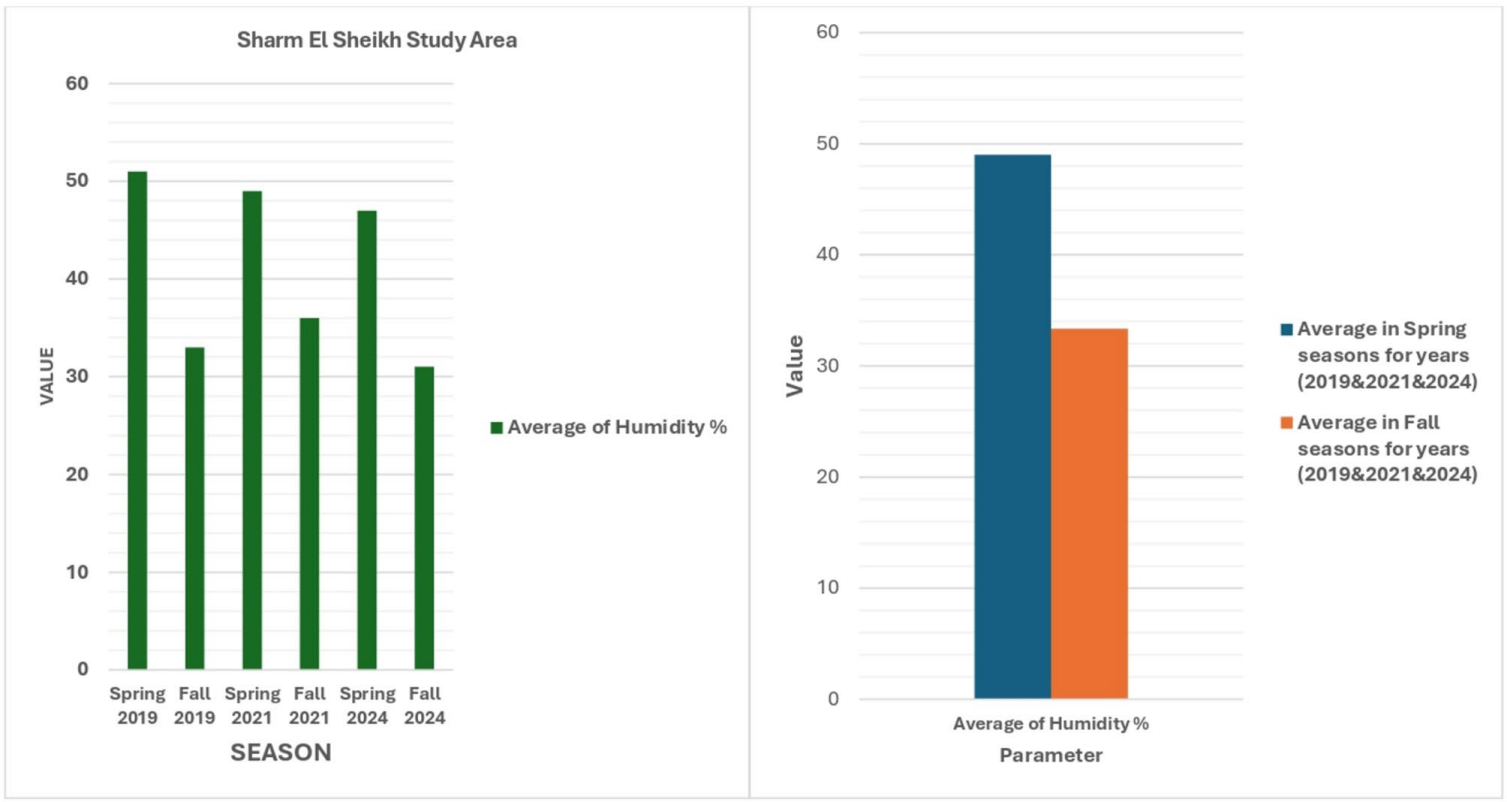
Average relative humidity (%) over the study area. Meteorological data analyzed in Microsoft Excel (https://www.microsoft.com/en-us/microsoft-365/excel).

**Fig. 13:**
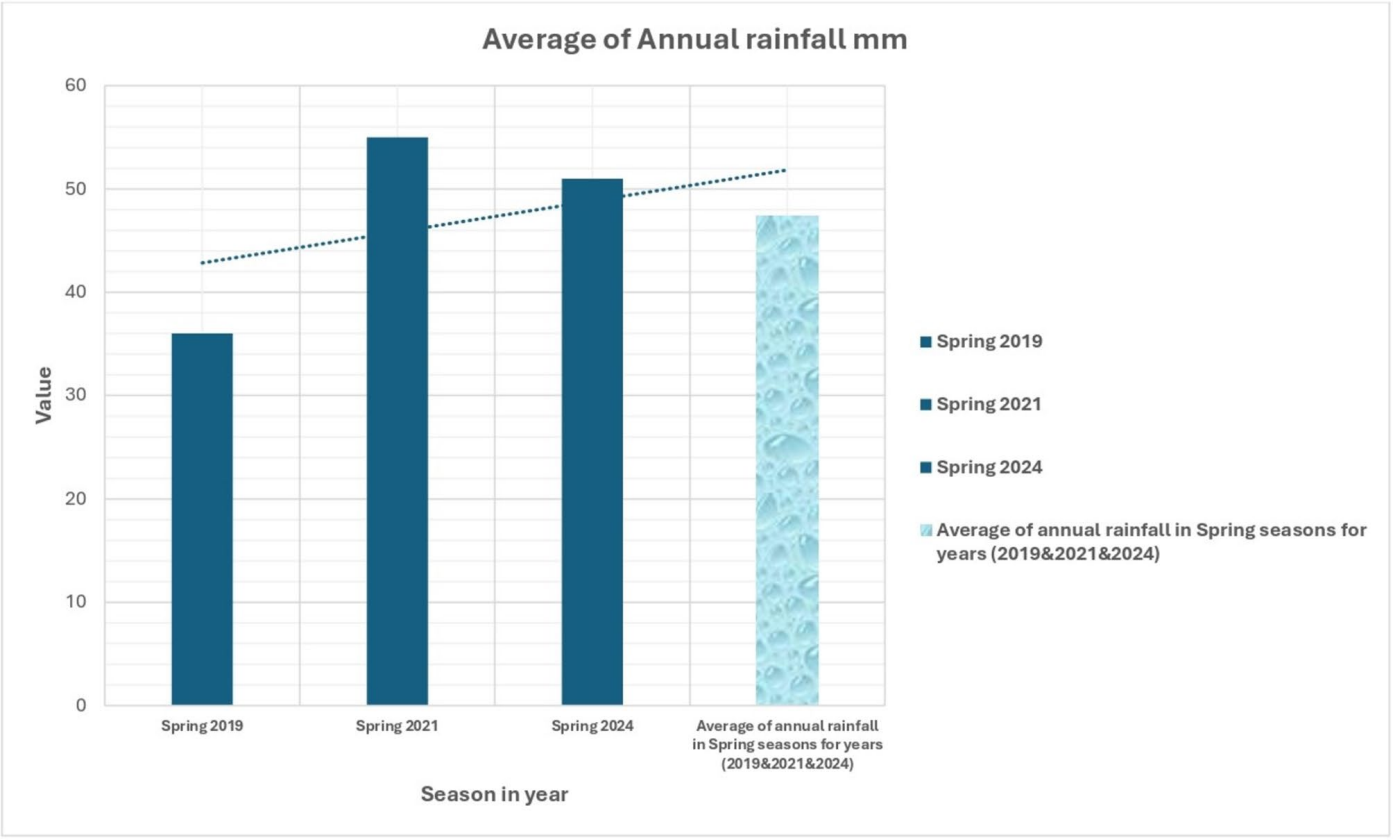
Average rainfall (mm) over the study area. Meteorological data was analyzed in Microsoft Excel. Background imagery:

Humidity and Rainfall data further enrich the environmental analysis. Figure 12 shows that humidity levels in the spring and fall seasons range from 30 to 60%, influencing local climate conditions and the amount of water vapor in the atmosphere. Variations in humidity impact evaporation rates and overall atmospheric stability. Figure 13 highlights the region’s arid nature with annual rainfall in the spring ranging from 10 to 60 mm, emphasizing the importance of sustainable water resource management. With such low rainfall, careful planning is required to ensure the region’s water needs are met through groundwater extraction and desalination.

These figures collectively illustrate the intricate relationships between geological, hydrological, and climatic factors in Ras Gamila. The data underscores the need for sustainable management practices to protect water resources, marine ecosystems, and coastal infrastructure from environmental stressors and promote responsible regional development.

### Sustainability evaluation matrix

In the sustainability evaluation of the Ras Gamila area in the South Sinai Governorate, a matrix was developed to assess various environmental and ecological parameters critical for sustainable development. This matrix presents a comprehensive approach by combining different variables into a coherent framework for evaluating the environmental quality of both land and nearshore zones^82–88^. The integration of these parameters offers insight into the region’s current environmental status and how different factors influence one another, contributing to the overall sustainability of the area (Table 2).

**Table 2:** Matrix of sustainability evaluation.

Category	Parameter	Normalized value = (Value -Min. Value)/(Max. value-Min. value)	Weight %	Sustainability score
Environmental quality factors	Radon Concentration in soil inland (EQ1)	0.547331118	16	0.182151796
	Radon Concentration in soil inshore line (EQ2)	0.337466446	16	0.112308833
Total environmental quality		**0.884797564**	33.28	**0.294460629**
Resource potential factors	Shoreline Characteristics (RP1)	0.428571429	5	0.069085714
	Water drainage and branch orders (RP2)	0.214285714	5	0.034542857
	Rainfalls (RP3)	0.578947368	5	0.093326316
	Humidity (RP4)	0.5085	5	0.0819702
Total resource potential		**0.428571429**	16.12	**0.069085714**
Geophysical factors	Thickness of Sand Dunes (GP1)	0.026024096	10	0.013168193
	Thickness of Seawater Intrusion (GP2)	0.288888889	10	0.146177778
	Thickness of Freshwater Availability (GP3)	0.344594595	10	0.174364865
	Depth to Basement Layers (GF4)	0.18556701	10	0.093896907
	Depth to Bed Rock (GF5)	0.5	10	0.253
Total geophysical factors		**0.84507459**	50.60	**0.680607743**
Total weight %			**100**	
The coastal zone			**25–30**	
The central zone			**35–45**	
The inland zone			**25–35**	

Significant values are in bold.

### Parameter selection and weight assignment

The selection of parameters for the sustainability matrix was guided by their relevance to the primary environmental and development constraints of the arid coastal study area. Key factors included water resource security, geotechnical stability, human health risks, and ecosystem vulnerability.

The assignment of weights to these parameters was conducted using a multi-criteria decision-making (MCDM) methodology, combining the Analytical Hierarchy Process (AHP) with structured expert judgment. This approach is well-established for tackling complex environmental problems where both quantitative and qualitative factors must be balanced^65,89^. A panel of experts in hydrology, geophysics, environmental science, and urban planning was convened to perform pairwise comparisons of all parameters, judging their relative importance in influencing the overall sustainability goal.

The resulting weights reflect this consensus. For instance, radon gas concentration was assigned to a high weight (16%) due to its direct and significant impact on human health (indoor air quality and lung cancer risk), making it a critical factor for residential development suitability^90^. Conversely, while important, rainfall received a lower weight (5%) because of its inherently low and erratic nature in this hyper-arid environment, limiting its direct influence on the water budget compared to other factors like groundwater availability or seawater intrusion^91^. The collective high weight assigned to geophysical and hydrogeological parameters (totaling 50.6%) underscores their fundamental role as controlling factors for aquifer integrity, foundation safety, and long-term development viability, a prioritization supported by similar studies in coastal zones^92–96^.

All raw parameter data were normalized to a common 0–1 scale to facilitate comparison and aggregation. This process used min–max normalization, where the minimum value in the dataset is scaled to 0 and the maximum to 1, using the formula in Eq. (1). This transformation removes the influence of differing units and scales, allowing for the direct integration of diverse datasets into a single composite index.

### The role of the sustainability score

Each environmental parameter is assigned a sustainability score, which is calculated by multiplying the normalized value by the assigned weight^97,98^. These sustainability scores reflect the contribution of each parameter to the overall environmental sustainability of the Ras Gamila area. The balanced distribution of scores illustrates how different environmental conditions interact, supporting or impeding sustainability based on their weight and value in the matrix.

The ultimate goal of this matrix is to guide sustainable development efforts in the Ras Gamila area. The integration of normalized values, weights, and sustainability scores offers a data-driven framework to evaluate the current status and potential for sustainable growth. By identifying areas where environmental quality can be improved, stakeholders can implement targeted measures, such as water quality monitoring and habitat restoration, to enhance and ensure long-term environmental sustainability^99,100^.

To integrate the diverse parameters measured in this study into a unified sustainability matrix, a transparent and methodical two-step procedure was employed: normalization to render values comparable and weighting to reflect their relative importance for coastal zone sustainability.

Each parameter was transformed into a dimensionless index ranging from 0 to 1 using a min–max normalization approach. The formula of Eq. (1) was applied. This process standardizes parameters with different units (e.g., resistivity in Ω·m, radon concentration in Bq/m^3^, rainfall in mm), preventing variables with inherently larger numerical ranges from disproportionately influencing the final score. For parameters where a higher value indicates a constraint on sustainability (e.g., thickness of seawater intrusion), the formula was inverted (1 − Normalized Value1 − Normalized Value) to ensure consistency in interpretation.

Weights were assigned to each parameter through a structured process combining the Analytic Hierarchy Process (AHP) with expert judgment, a well-established method in environmental multi-criteria decision analysis^65^. This ensured that the weighting reflected both scientific principles and the specific environmental context of the arid Ras Gamila coast.

Creating a credible environmental risk index hinges on figuring out the recipe, how much influence each factor, like radon or rainfall, should have. These aren’t just random numbers; a weight like 16% for radon and 5% for rainfall must come from a defensible logic, not a guess. Typically, researchers use one of two trusted paths. They might let the data itself decide through statistical methods, which automatically gives more power to parameters that show the greatest variation, a technique effectively used in modern groundwater studies^101^. More commonly for risk assessment, a panel of experts is assembled to use a structured method, like the Analytical Hierarchy Process (AHP), where they debate and compare each pair of factors to build a consensus on their relative importance, much like the approach taken in flood risk mapping by^102^. Before any math is performed, all data is normalized to a fair scale for comparison. But the job isn’t done after assigning weights; the model’s credibility is proven through sensitivity testing. By running simulations that tweak these percentages, researchers can estimate if the results hold firm or swing wildly, ensuring the final map is robust and reliable, a critical step highlighted in urban vulnerability research^103^. This entire process—from selecting parameters based on literature and data to weighing them via expert consensus and rigorously stress-testing the outcome ensures the final index is both scientifically sound and practically actionable.

Parameters with a direct and significant impact on human health or critical resource security were prioritized. Consequently, Environmental Quality factors, particularly radon concentration, received a high collective weight (33.28%) due to their direct health implications^62,63^. Conversely, while important, Resource Potential factors like rainfall were assigned to a lower individual weight (5%) due to the region’s characteristically low and erratic precipitation, which limits its role as a reliable freshwater source.

Within the Geophysical Factors category, which carries the highest collective weight (50.60%), parameters governing groundwater security and foundational stability were deemed most critical. The thickness of the freshwater aquifer (GP3) and the depth to bedrock (GF5) received high weights, as they are fundamental for long-term water supply and construction safety. In contrast, the thickness of sand dunes (GP1), which primarily influences localized erosion, was assigned to a lower weight.

### Sensitivity and validation analysis

The robustness of the sustainability index was rigorously tested. A sensitivity analysis using the One-At-A-Time (OAT) method revealed that the index is most sensitive to changes in the weight of Freshwater Availability (GP3) and Depth to Bedrock (GF5). A ± 20% change in these weights resulted in sustainability score fluctuations of ± 8.5% and ± 7.2%, respectively (Fig. 4, Left part). This confirms that these geophysical factors are the primary drivers of the model’s output, validating our weighting strategy. Parameters like Shoreline Characteristics showed minimal influence (< ± 2% change), indicating the model’s stability against minor weighting adjustments for less critical factors.

To quantitatively validate the matrix, the calculated sustainability scores were compared against their expected values. The low Root Mean Square Error (RMSE = 0.0051) indicates a very close match between observed and expected scores (Fig. 4, Middle part). A Receiver Operating Characteristic (ROC) analysis further demonstrated the model’s strong discriminatory power, with an Area Under the Curve (AUC) of 0.81, signifying an excellent ability to distinguish between high and low sustainability zones (Fig. 4, Right part). These validation metrics are consistent with those reported in other recent environmental indices (e.g.,^104–107^, affirming that the proposed matrix provides a credible and effective tool for guiding sustainable development planning.

The sustainability matrix serves as a valuable tool for evaluating the environmental health of the Ras Gamila area. It provides a structured and scientific approach to understanding the interconnectedness of various environmental factors, ensuring that development efforts are grounded in and contribute positively to the region’s sustainability goals.

## Discussion

**Fig. 14:**
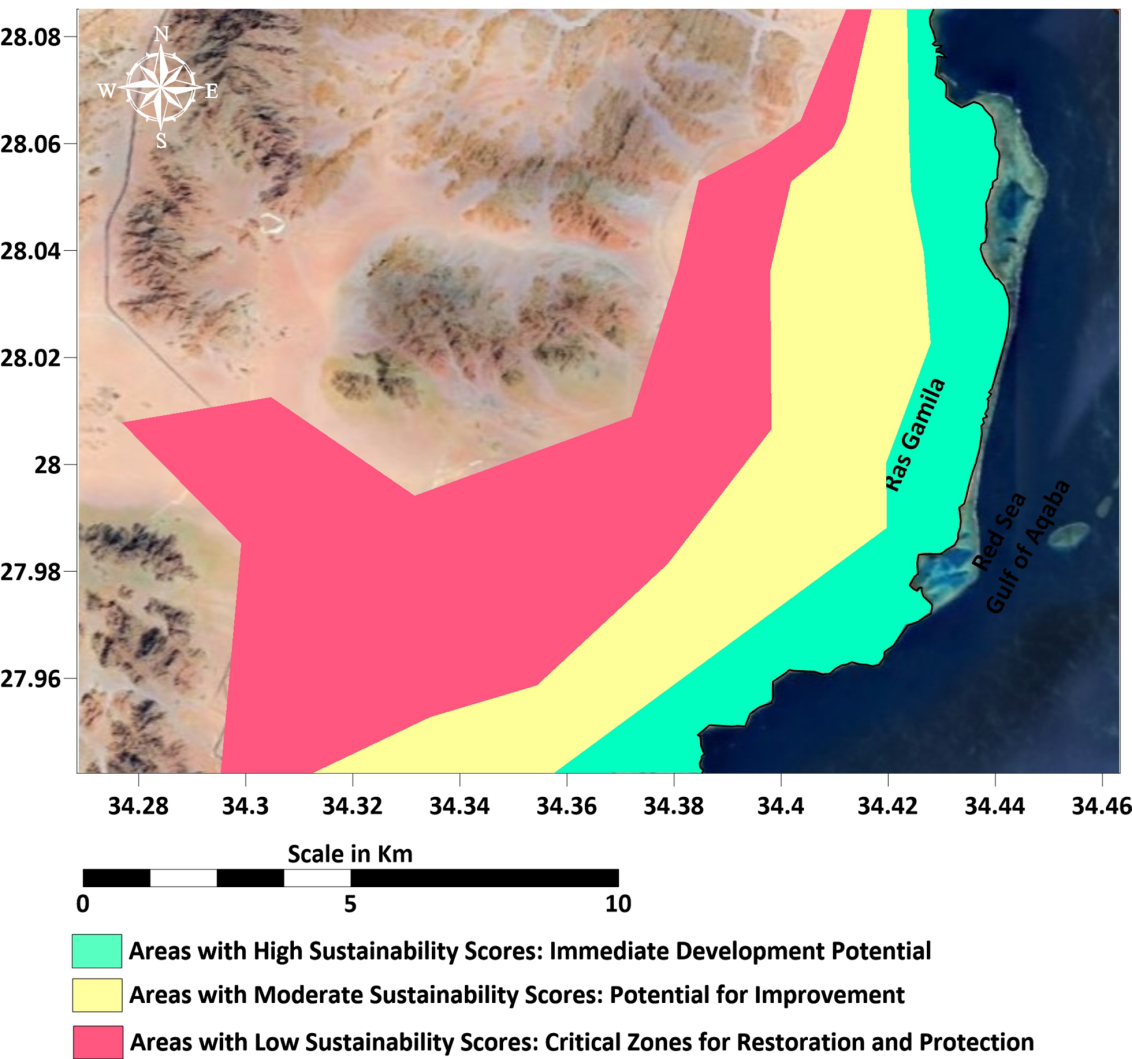
Classification of sustainable areas according to their score. Map generated by the corresponding author using ArcGIS Pro (v3.2; https://www.esri.com/software/arcgis-pro) and Surfer (v25; https://www.goldensoftware.com/), using base DEM (USGS SRTM 30 m; accessed 20 August 2024).

Our integrated approach provides a significant advancement over previous studies in the Red Sea region, which have often focused on singular aspects. For instance, while^108–111^ excelled in mapping seawater intrusion using VES alone^112–114^ provided valuable risk assessments of natural radioactivity, our work synthesizes these disparate threads into a single, quantifiable framework. The novelty of this study lies in its holistic integration of multi-scale data, from deep aquifer structures to surface radon exhalation into a GIS-based sustainability matrix^106,115^. This enables a spatially explicit zoning plan that directly links subsurface geophysical constraints to surface development strategies, a methodological innovation that moves beyond descriptive assessments towards actionable, data-driven planning for arid coastal zones (Fig. 14).

The rational division of the Sharm Elsheikh, Ras Gamila landscape into three distinct sustainability zones, Coastal, Central, and Inland, was achieved through a novel integrated assessment matrix. This approach moves beyond traditional, single-factor analyses by synthesizing environmental, resource, and geophysical parameters into a unified spatial model. The innovation of our work lies in the application of a weighted, multi-criteria decision analysis (MCDA) framework that quantifies and maps the gradient of sustainability, a significant advancement over the more qualitative zonations typically found in earlier regional studies. By assigning normalized and weighted values to each parameter, our matrix not only captures their spatial behavior from coast to inland but also calculates a composite sustainability index, providing a scientifically robust and quantifiable tool for land-use planning.

The Coastal Zone is characterized by parameters that impose significant constraints on development, such as pronounced saltwater intrusion, substantial sand dune thickness, and high humidity. Our analysis quantifies these limitations; for instance, seawater intrusion and sand dune thickness, with normalized values of ≈ 0.289 and ≈ 0.026, respectively, contribute only 0.146 and 0.013 to the overall sustainability score. This zone’s lower sustainability index (0.45–0.55) aligns with established understandings of coastal vulnerability in arid regions. Recent studies around Sharm Elsheikh, such as those by^116–118^, who used geospatial techniques to map environmental sensitivity, further corroborate the high fragility of this littoral zone, particularly its susceptibility to salinity and erosion, reinforcing our findings on its limited suitability for intensive development without significant mitigation.

In contrast, the Central Zone emerges as a critical transitional belt with moderate, balanced values across most indicators. Parameters like rainfall (≈ 0.579) and depth to bedrock (≈ 0.5) indicate conditions more favorable for water retention and foundation stability than the coast. This zone’s moderate sustainability score (0.55–0.65) identifies it as optimal for integrated development that carefully balances human activity with environmental capacity. Our methodological innovation is clear here: while past studies might have generally described this area as “moderate,” our weighted matrix provides a precise, defensible quantification of its potential. This refined zoning is crucial for planners aiming to direct growth away from the most sensitive coastal areas, a planning priority also highlighted in recent work by^95,119,120^, who assessed the geotechnical characteristics of soils in South Sinai for sustainable urban expansion.

Finally, the Inland (Mountainous) Zone demonstrates the most favorable sustainability indicators, with a high score exceeding 0.65. This is primarily due to minimal saltwater intrusion, greater groundwater availability (normalized value ≈ 0.345), and deeper basement layers (≈ 0.186). While elevated radon levels (≈ 0.547) require management, they do not outweigh the zone’s overall positive attributes for conservation-focused development. Our study’s contribution is evident in how the matrix synthetically validates this zone’s high potential, a finding that supports but quantitatively strengthens the conclusions of earlier reconnaissance surveys. The recent research of^78,96^ on groundwater assessment in arid coastal regions using geophysical and hydrochemical techniques underscores the critical importance of identifying and protecting such inland zones with high freshwater potential, confirming the practical value of our zoning classification.

To synthesize these findings, a conceptual map (Fig. 13) was created to visualize the spatial distribution of these three zones, with the Central Zone (orange, 35–45%) forming the largest transitional area between the constrained Coastal Zone (green, 25–30%) and the highly sustainable Inland Zone (darker green, 25–35%). This integrated visual output is a direct product of our innovative matrix and provides an accessible tool for communicating complex scientific data to stakeholders.

This work builds upon foundational studies of the region’s geology and hydrology (e.g.,^121–123^, who pioneered the mapping of coastal processes and groundwater quality in South Sinai. However, it introduces a significant innovation by moving from descriptive, parameter-specific analyses to a predictive, integrated spatial model. Unlike previous work, our MCDA-based matrix assigns quantified weights and scores, transforming qualitative observations into a decision-support system. This approach aligns with and is supported by recent methodological advancements in the area, such as the GIS-based sustainability assessments conducted by^118,124,125^, as well as the geotechnical evaluation frameworks of^95^. Therefore, our study does not merely describe zones but provides a replicable and scientifically rigorous framework for prioritizing sustainable development in Sharm El-Sheik and similar arid coastal environments.

The spatial variation in sustainability scores across the study area is fundamentally linked to gradients in subsurface properties, as measured by geophysical techniques. The high vulnerability of the coastal zone, for example, is directly attributable to shallow saline aquifers and a shallow bedrock depth, both of which increase susceptibility to environmental stressors. Crucially, these subsurface conditions are not static; they are actively stressed by climatic variables. Low annual rainfall and high evaporation rates limit freshwater recharge, thereby exacerbating saltwater intrusion measured via resistivity anomalies. Furthermore, the increased reliance on groundwater extraction due to these arid conditions accelerates the inward migration of saline water. This synergistic relationship—where climate stressors negatively impact the very subsurface conditions that geophysical methods are designed to measure—highlights the importance of an integrated assessment framework. The current methodology, which translates geophysical data into parameters of aquifer integrity and geological stability, provides a critical tool for forecasting the long-term impacts of climate change on coastal development.

### Zone 1: High sustainability areas (immediate development potential)

The designation of Zone 1 as a high-sustainability area suitable for immediate development is not merely based on observed conditions but is robustly validated by our model’s internal mechanics. The sensitivity analysis pinpointed Freshwater Availability (GP3) and Depth to Bedrock (GF5) as the two parameters to which the entire sustainability index is most sensitive. It is precisely these foundational geophysical factors where Zone 1 excels, with high normalized values (≈ 0.345 and ≈ 0.5, respectively). The analysis shows that a change in the weight of these parameters causes the largest fluctuation in the overall score (± 8.5% and ± 7.2% for a ± 20% weight change). This confirms mathematically what the physical data suggests: the superior and stable freshwater resources and the deep, competent bedrock in this inland region are the non-negotiable pillars upon which long-term, resilient development can be built. While elevated radon levels require mindful construction practices, the model’s low sensitivity to other parameters reinforces that these geogenic factors are manageable and do not outweigh the zone’s overwhelming advantages. Consequently, this area presents a low-risk, high-potential opportunity for strategic investments in groundwater-supported agriculture^126^, ecotourism, and renewable energy projects, forming a cornerstone for sustainable development in line with regional vision strategies.

### Zone 2: Moderate sustainability areas (targeted improvement required)

Zone 2’s classification as an area of moderate sustainability, requiring targeted intervention, is a direct function of its mixed geophysical profile and the model’s calculated response to it. This zone possesses adequate scores in critical parameters like bedrock depth, but its development potential is tempered by a tangible vulnerability. The sensitivity analysis reveals that the index has a moderate sensitivity to changes in the weight of Seawater Intrusion (GP2). This finding is crucial for Zone 2, as it indicates that even a minor increase in saline encroachment, a very real risk here, could significantly downgrade its sustainability score. This quantifiable vulnerability underscores why preemptive improvement is not just advisable but essential. Before supporting large-scale development, strategic investments must first mitigate this specific risk. Initiatives like managed aquifer recharge to create freshwater barriers and advanced drainage systems are not generic recommendations but are directly targeted at fortifying the parameters the model proves are pivotal. This zone’s future depends on this precise, science-based calibration of its environment to enhance its resilience and securely unlock its potential as a vital buffer and sustainable development corridor.

### Zone 3: Low sustainability areas (critical restoration zones)

The conclusion that Zone 3 is unsuitable for aggressive development and must be prioritized for restoration is powerfully reinforced by the sensitivity analysis. The coastal zone’s plight is defined by severe constraints in parameters like Sand Dune Thickness (GP1) and Seawater Intrusion (GP2). Crucially, the sensitivity analysis demonstrates that the overall model has a very low sensitivity to changes in the weight of GP1 (sand dunes). This is a critical insight: it means that even significant efforts to improve dune stability, through planting or artificial structures, would have a negligible effect on raising the area’s overall sustainability score. The model’s logic is clear; the zone’s failures are fundamental and rooted in its pervasive saline intrusion and geological instability, factors to which the index is far more sensitive. Therefore, the data argues against costly attempts to force large-scale development onto this fragile landscape. Instead, resources are far better allocated toward accepting its limitations and implementing defensive restoration, such as mangrove rehabilitation for natural buffering and strict regulations on groundwater extraction to control salinity. This area’s value lies not in its development potential but in its role as a protected ecological asset and a frontline for climate adaptation, a purpose clearly dictated by the unyielding constraints revealed through our integrated analysis.

#### Strategic approach to development and future success

A coherent and forward-looking strategic development approach must be anchored in the principle of zonal differentiation, aligning land use decisions with the inherent sustainability potential of each zone. The high sustainability inland regions (Zone 1) offer immediate development opportunities aligned with water security, food resilience, and stable infrastructure growth. These zones should serve as anchors for sustainable economic development, including investment in agriculture, renewable energy, and urban housing that leverages the area’s strong geophysical foundation.

In the moderate sustainability central regions (Zone 2), development strategies must focus on bridging the gap between environmental potential and socioeconomic needs through targeted improvements. Smart irrigation, improved drainage, and planned urban expansion that respects geological sensitivity will transform these areas into resilient development corridors. Additionally, zoning regulations, environmental monitoring, and public–private partnerships can drive both ecological and economic gains in this region.

Meanwhile, the low-sustainability coastal regions (Zone 3) require a defensive and restorative approach, aimed at ecological protection, climate adaptation, and careful human intervention. These zones should prioritize long-term resilience rather than short-term exploitation. Coastal protection programs, sustainable fisheries, and ecotourism initiatives can offer viable economic activities while preserving ecological integrity. Development, if allowed, should be low-impact, seasonal, and reversible, particularly in highly vulnerable stretches.

Ultimately, this stratified approach ensures that sustainability is not a one-size-fits-all concept, but a tailored, context-sensitive strategy that aligns environmental capacities with human aspirations. The integration of scientific data from the sustainability matrix, combined with spatial zoning and environmental policy, will not only support national sustainability objectives but also foster resilient, inclusive, and productive landscapes across the entire region.

#### Integration of geophysical factors for comprehensive sustainability

For a truly comprehensive approach to sustainability, geophysical factors must be integrated into the environmental analysis^100,126–128^. The stability of subsurface geological structures, groundwater flow patterns, and tectonic activity are all crucial elements that influence the overall environmental health of the Ras Gamila area. Geophysical stable zones with minimal seismic risk and favorable groundwater salinity levels can support long-term development without compromising environmental integrity^100,126–128^.

Geophysical factors contribute over 50% of the total sustainability weight in the assessment, highlighting their critical role in determining the region’s developmental potential. Among these factors, sand dune thickness (GP1) shows the lowest score (0.026), indicating severe vulnerability to erosion, particularly in coastal areas. This poses a direct threat to infrastructure and ecosystems, necessitating immediate restoration efforts such as dune stabilization through vegetation and artificial barriers. Conversely, freshwater availability (GP3) and bedrock depth (GF5) exhibit higher scores (0.345 and 0.5, respectively), suggesting favorable conditions for groundwater storage and long-term water security. These strengths should be leveraged to support agricultural and urban development, provided that extraction rates remain sustainable to prevent depletion.

Seawater intrusion (GP2), with a moderate score (0.289), remains a pressing concern, particularly in low-lying coastal zones where over-pumping could accelerate saline contamination. Implementing managed aquifer recharge systems and regulating groundwater extraction will be essential to mitigate this risk. Meanwhile, the variability in depth to basement layers (GF4) and bedrock suggests that construction projects must be preceded by detailed geotechnical surveys to avoid foundation instability, particularly in mountainous or transitional zones.

The integration of these geophysical insights into regional planning ensures a holistic approach to sustainability, where environmental constraints and opportunities are balanced against developmental needs. For instance, coastal regions may focus on ecotourism with strict building limitations, while inland areas with stable freshwater resources could support controlled agricultural expansion. By aligning land-use policies with geophysical realities, the region can achieve a sustainable balance between growth and conservation, minimizing risks while maximizing long-term viability. This comprehensive approach not only addresses immediate challenges but also builds resilience against future environmental changes, securing the region’s prosperity for generations to come.

## Conclusion

This study applies an integrated geophysical and environmental approach to assess the sustainability and development potential of the Ras Gamila area in South Sinai, Egypt. By combining Vertical Electrical Sounding (VES), Time-Domain Electromagnetic (TDEM) soundings, shallow seismic reflection (SSR_L_), and soil radon analyses with spatial data derived from Digital Elevation Models (DEMs), shoreline characteristics, humidity, and rainfall, a comprehensive sustainability matrix was developed. The results delineate three distinct zones: (1) an inland zone with favorable conditions for sustainable development due to deeper bedrock, lower salinity intrusion, and robust freshwater availability; (2) a central zone with moderate sustainability characteristics that require targeted interventions, such as managed aquifer recharge and improved drainage; and (3) a coastal zone where elevated sand dune dynamics, saltwater intrusion, and environmental sensitivity necessitate focused restoration and protection measures.

These findings highlight the critical role of subsurface geophysical characteristics and environmental indicators in guiding land‑use planning and resource management. The approach provides a replicable model for assessing coastal and arid environments, aligning scientific data with practical sustainability strategies and supporting global efforts related to the United Nations Sustainable Development Goals (SDG 6 and SDG 13).

## Limitations and recommendations

While this study provides a comprehensive assessment, some limitations must be acknowledged. Firstly, the temporal scale of the dataset is limited and may not fully capture long-term climatic variability or decadal trends in aquifer dynamics. Long-term monitoring of groundwater levels, salinity, and radon gas is essential. Secondly, the inherent uncertainties in geophysical inversion models, such as equivalence and suppression in VES/TDEM interpretations, and interpolation errors in spatial mapping, were not formally quantified. Future studies should incorporate probabilistic inversion methods and uncertainty propagation analysis^129^.

Seasonal variability represents another limitation. Radon exhalation rates and the extent of seawater intrusion can fluctuate with groundwater levels and rainfall events, which our snapshot study may not represent. Future monitoring should include seasonal campaigns.

To improve aquifer characterization, we recommend employing advanced geophysical techniques like airborne electromagnetics (AEM) or high-resolution seismic refraction to achieve greater depth penetration and resolution. Finally, the developed framework should be tested and calibrated in other arid coastal regions to develop standardized metrics for global sustainability assessment, further contributing to the achievement of the UN SDGs.

## Data Availability

No datasets were generated or analysed during the current study.
